# High content organelle trafficking enables disease state profiling as powerful tool for disease modelling

**DOI:** 10.1038/sdata.2018.241

**Published:** 2018-11-13

**Authors:** Arun Pal, Hannes Glaß, Marcel Naumann, Nicole Kreiter, Julia Japtok, Ronny Sczech, Andreas Hermann

**Affiliations:** 1 Division of Neurodegenerative Diseases, Department of Neurology, Technische Universität Dresden, Dresden, Germany; 2 German Center for Neurodegenerative Diseases (DZNE) Dresden, Dresden, Germany; 3 Center for Molecular and Cellular Bioengineering, Technische Universität Dresden, Dresden, Germany; 4 Center for Regenerative Therapies Dresden (CRTD), Technische Universität Dresden, Dresden, Germany

**Keywords:** Membrane trafficking, Image processing

## Abstract

Neurodegenerative diseases pose a complex field with various neuronal subtypes and distinct differentially affected intra-neuronal compartments. Modelling of neurodegeneration requires faithful *in vitro* separation of axons and dendrites, their distal and proximal compartments as well as organelle tracking with defined retrograde *versus* anterograde directionality. We use microfluidic chambers to achieve compartmentalization and established high throughput live organelle imaging at standardized distal and proximal axonal readout sites in iPSC-derived spinal motor neuron cultures from human amyotrophic lateral sclerosis patients to study trafficking phenotypes of potential disease relevance. Our semi-automated pipeline of organelle tracking with FIJI and KNIME yields quantitative, multiparametric high content phenotypic signatures of organelle morphology and their trafficking in axons. We provide here the resultant large datasets to enable systemic signature interrogations for comprehensive and predictive disease modelling, mechanistic dissection and secondary hit validation (e.g. drug screens, genetic screens). Due to the nearly complete coverage of analysed motility events, our quantitative method yields a bias-free statistical power superior over common analyses of a handful of manual kymographs.

## Background & Summary

The role of neurite trafficking defects in the pathophysiology of neurodegenerative diseases is a hotly debated issue^1–8^. Such perturbed transport logistics detrimentally impact on various essential cellular processes like signal transmission, gene regulation, nutritional metabolism, proteasome function, energy supply and distal protein biosynthesis, eventually leading to compromised axonal pathfinding, diminished neuronal plasticity and neurodegeneration^1–8^.

Amyotrophic lateral sclerosis (ALS) is a fatal, incurable motor neuron disease characterised by muscle denervation, demyelination and loss of spinal and cortical motor neurons (MNs) causing progressive muscle paralysis^9^. One important hallmark is the retrograde retraction and dying back of the axon from the neuromuscular junction^10–12^. In our recent publication^6^, we recapitulated these dying back processes *in vitro* using iPSC-derived spinal motor neurons from familiar ALS patients carrying a mutation in FUS, eventually resulting in neurodegeneration. Moreover, we observed severe distal trafficking defects of mitochondria and lysosomes in axons clearly preceding the onset of first structural damage, i.e. signs of dying back. Remarkably, the early rescue of these distal organelle trafficking defects through treatment with promising drug candidates completely abrogated the premature die back and neurodegeneration of ALS neurons, thereby suggesting that early restoration of premanifest disease hallmarks provides novel therapeutic intervention points in ALS. As for other mutated genes causing ALS, we recently observed trafficking defects in TDP43 mutants^3,13^ distinct from FUS, i.e. a more ‘global’ phenotype occurring in the proximal axon parts as well that developed more slowly during the aging of MNs in culture with a distinct response to rescue candidates^3^.

**Figure 1:**
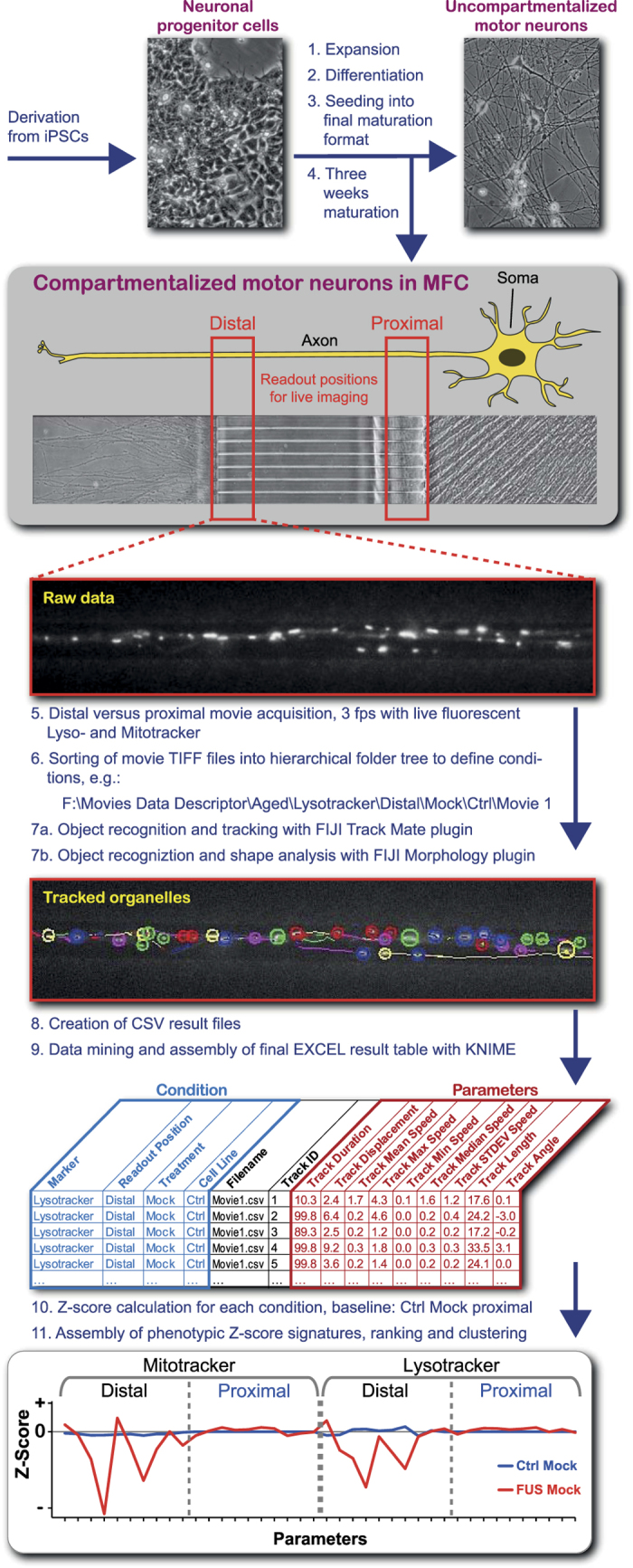
Schematic workflow of live multiparametric HC profiling of spinal motor neurons (MN) compartmentalized in Zona microfluidic chambers (MFC)s. Inducible pluripotent stem cells from human ALS patients (iPSCs) were differentiated *via* neuronal progenitor cells (NPCs) for final maturation to spinal motor neurons (MNs) in Zona microfluidic chambers (MFCs) to obtain compartmentalized cultures with a defined distal – to – proximal axon alignment in microchannels. Live imaging was performed at strictly standardized readout positions to obtain distal *versus* proximal organelle trafficking data with Mito- and Lysotracker. Resultant movies were sorted into a hierarchical folder tree to define meta conditions for automated dynamic organelle tracking and static morphology analysis in FIJI and subsequent data mining and result assembly in KNIME. Deviation from proximal Ctrl Mock was expressed as Z-score for a total of 40 parameters to obtain a HC signature as comprehensive quantitative descriptor for phenotypic deviations in diseased and drug/RNAi – manipulated states. Finally, entire signatures were ranked and pair-wisely agglomerated by hierarchical clustering.

In light of the above findings, disease modelling of axonal trafficking appears powerful in dissecting disease mechanisms and drug testing. In our recent publication^6^, we used organelle mean speed and track displacement as analytical live imaging readout parameters to document distal trafficking defects in axons. However, for accurate disease modelling we wished to evolve our original method further for a more ambitious disease profiling. Therefore, we established standardized live readout positions at distal and proximal positions in axons (Fig. 1) cultured in Xona RD900 microfluidic chambers (MFC) to enable their required compartmentalization and defined orientation (antero- *versus* retrograde). We verified the absence of dendrites in our readout windows by further immunostainings for MAP2 and SMI32 (Axon Specificity.jpg, Data Citation 1). By analysing the acquired movies in a semi-automated pipeline with the embedded FIJI Track Mate plugin for dynamic organelle tracking and particle analyser to model static organelle morphology, we achieved an analytical coverage of nearly all organelles (Tracking Illustration.jpg, Data Citation 1) yielding multiple phenotypic descriptors of high sensitivity. These comprised nine dynamic tracking parameters (Fig. 1, Dynamic Tracking Parameters.xlsx, Data Citation 1) and one static morphology parameter (Fig. 1, Static Morphology Parameters.xlsx, Data Citation 1). By combining the data from both the distal and proximal readout positions (Fig. 1) and both live trackers (Mito- and Lysotracker^3,6^), we obtained a comprehensive phenotypic HC signature of 40 quantitative parameters expressed as Z-score deviations from proximal wild type control baseline (Fig. 1). Our HC profiling is powerful for rigorous characterisation of disease states and their underlying mutations as well as drug and feasible RNAi interrogations for mechanistic and therapeutic studies.

**Figure 2:**
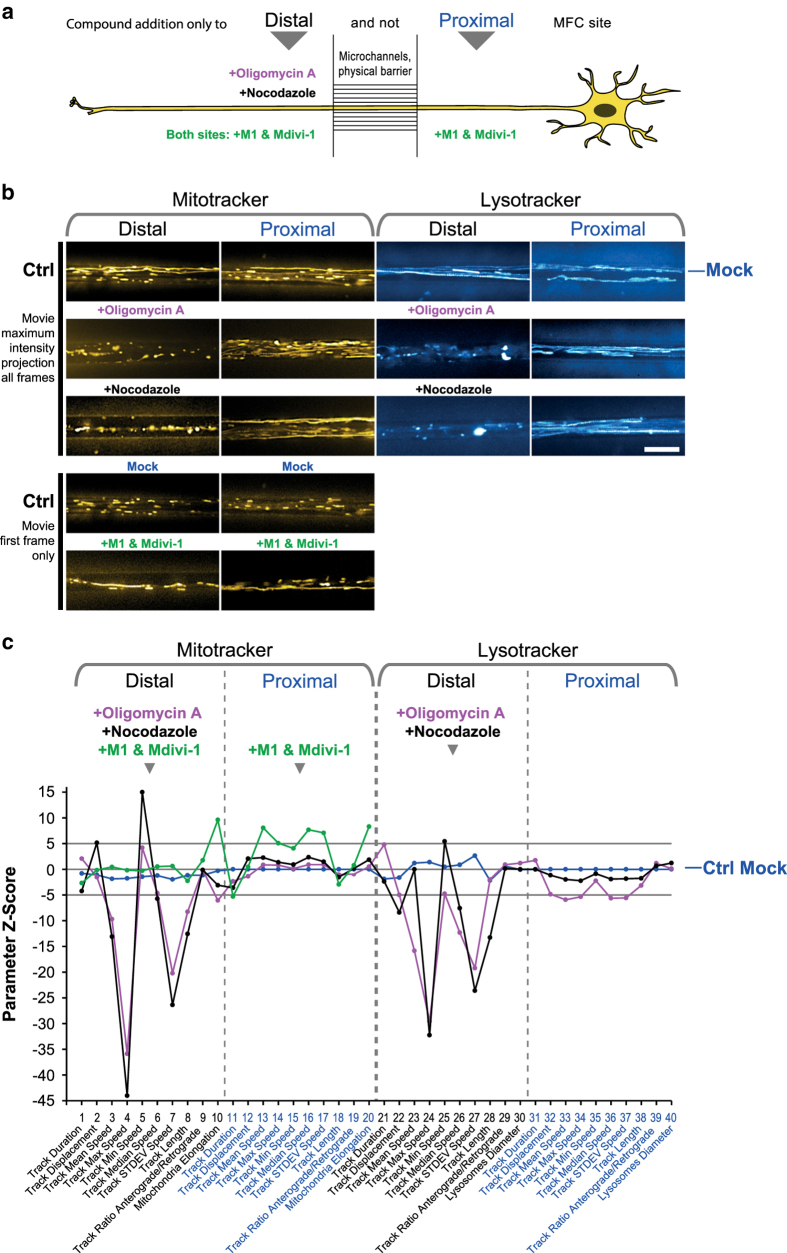
Validation of multiparametric HC profiling through drug–mediated alterations of organelle trafficking and shape in control MNs. **(a)** Schematic live set up of MNs in Zona MFCs. The central microgroove of channels formed a physical barrier between the distal (left) and proximal (right) site where the somas were seeded. Only axons could penetrate the microchannels. The tightness of the microgroove barrier enabled site–specific drug application: oligomycin A (pink) and nocodazole (black) were added exclusively to the distal MFC site to locally disrupt distal organelle trafficking, whereas a mixture of M1 & Mdivi-1 (green) was applied to either site to promote fusion and elongation of mitochondria. **(b)** Maximum intensity projections of movie raw data acquired live with mitotracker (left) and lysotracker (right) at the distal (left) versus the proximal (right) microchannel readout position (Fig. 1). Note how distal application of oligomycin a (pink) or nocodazole (black) disrupted distal organelle motility whereas proximal motility remained unaltered, indicating local action of both drugs exclusively at the distal MFC site.. As for M1 & Mdivi-1 mix, note how application to either MFC site led to extended elongation of mitochondria (only first movie frame shown here). Scale bar = 10 μm. **(c)** Multiparametric HC Z-score signatures obtained on Ctrl MNs after treatment with oligmycin A (pink), nocodazole (black) or a M1 & Mdivi-1 mix. Site – specific drug application was as indicated in the header and (a). Z-scores show deviations from proximal Ctrl Mock (blue baseline), Z-score above 5 and below -5 (grey lines) were considered as clearly significant deviations from Ctrl Mock (blue). **(d)** Hierarchical cluster dendrogram of entire signatures shown in (c). The hierarchical Z-score (ordinate) indicates the deviation of entire signatures from each other and is not to be mistaken with the individual parameter Z-scores in (c). **(e)** Hierarchical cluster dendogram of partial signatures comprising either only all distal or proximal parameters (Mitotracker and Lysotracker, respectively) to compare site – specific phenotypes.

**Figure 3:**
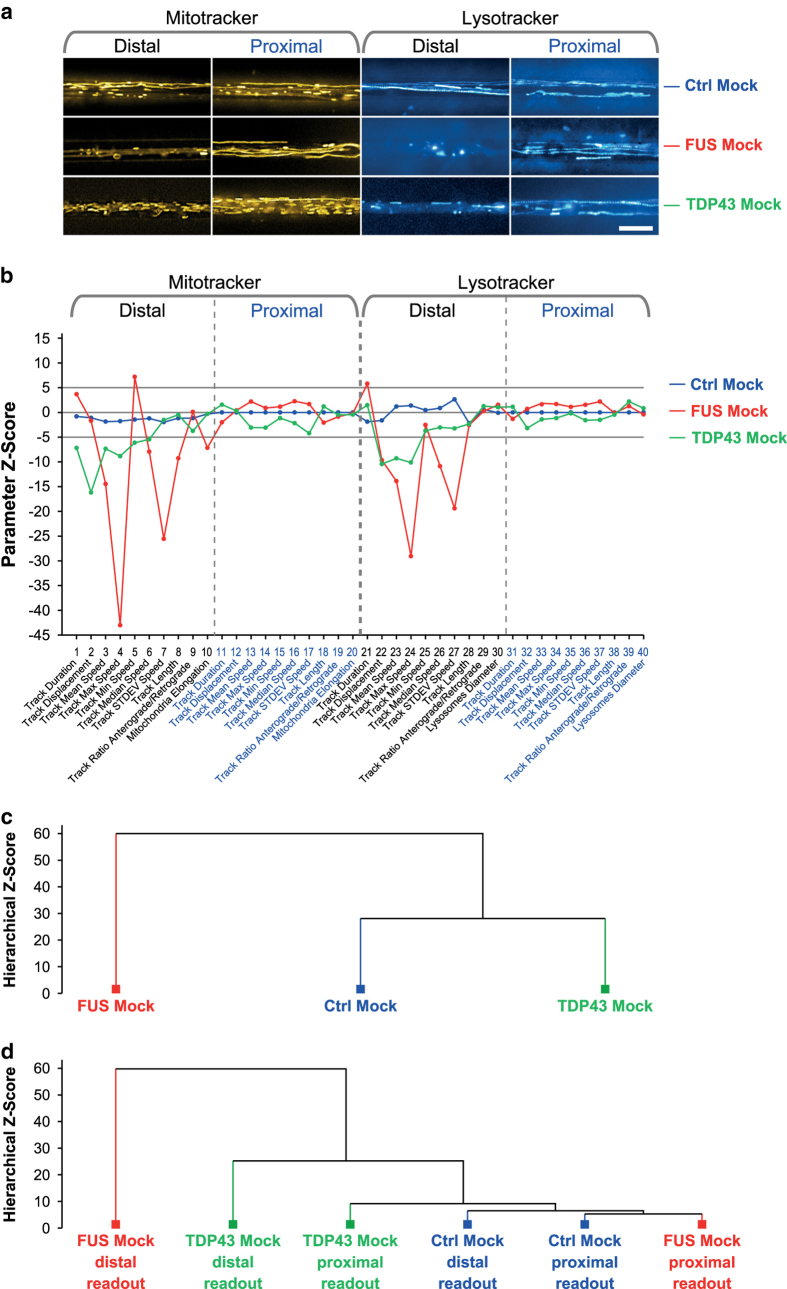
Validation of multiparametric HC profiling through disease models with confirmed axonal trafficking defects. **(a)** Maximum intensity projections of movie raw data acquired live with mitotracker (left) and lysotracker (right) at the distal (left) versus the proximal (right) microchannel readout position (Fig. 1). Note the virtual organelle arrest in the FUS (red) and TDP43 (green) mutants at the distal MFC readout site (loss of straight trajectories compared to Ctrl Mock above) whereas proximal motility remained unaltered. Scale bar = 10 μm. **(b)** Multiparametric HC signatures corresponding to (a). Note the nearly unaltered trafficking of both mutants at the proximal MFC readout site as compared to Ctrl Mock (blue) as opposed to strong negative parameter deviations at the distal site in FUS (red) distinct from the more modest phenotype of TDP43 (green). **(c)** Hierarchical cluster dendrogram of entire signatures in (b). Note how the modest TDP43 mutant (green) clustered with Ctrl Mock (blue) against the phenotypically more distinct FUS mutant (red). **(d)** Hierarchical cluster dendrogram of partial signatures comprising either only all distal or proximal parameters to compare site – specific phenotypes. Note how both physiological Ctrl Mock parts (blue, distal and proximal) clustered closely with the proximal FUS Mock part (red) on the right due to the close physiological trafficking state whereas the drastic organelle arrest in the distal FUS Mock part on the far left (red) was highly distinct to the physiological parts at the proximal site. TDP43 showed some moderate deviation in its proximal part (green) from the physiological clusters (Ctrl Mock, blue, and FUS Mock proximal, red) and a clear deviation in its distal part (green), albeit less drastic than FUS Mock (red).

**Figure 4:**
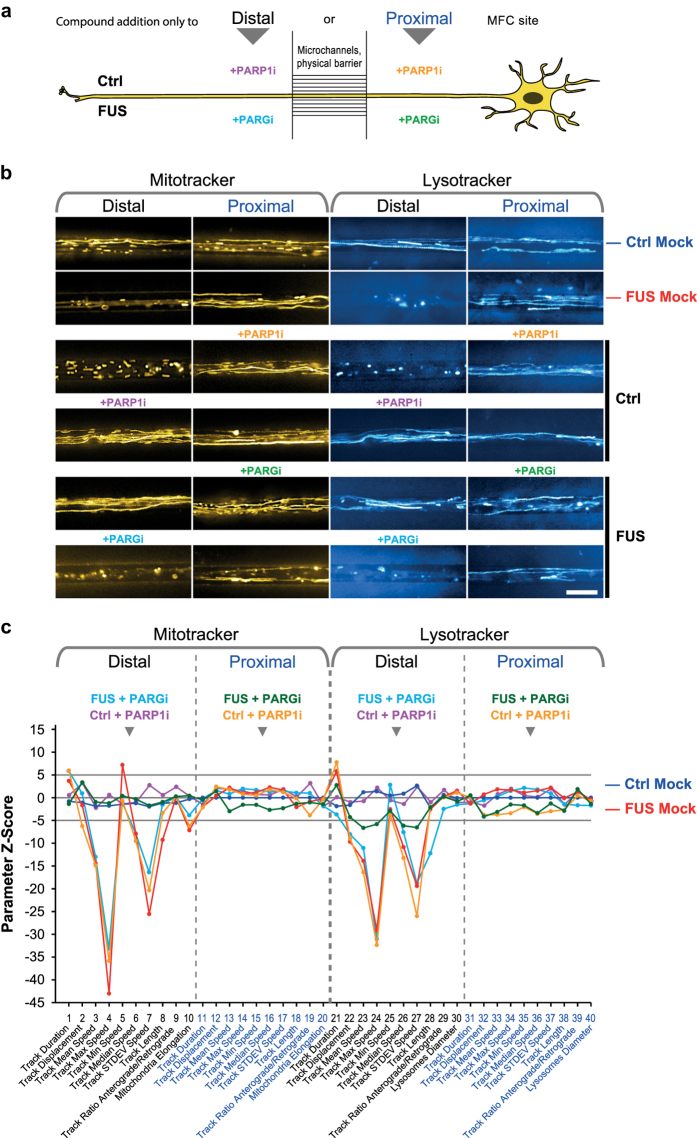
HC imaging as powerful tool for pathway or drug validation. **(a)** Schematic live set up of MNs in Zona MFCs. PARP1i was added to Ctrl MNs exclusively either to the distal (pink) or proximal (orange) MFC site while PARGi was added to FUS MNs exclusively either to the distal (pale blue) or proximal (green) MFC site to discriminate between local and remote action. **(b)** Maximum intensity projections of movie raw data acquired live with mitotracker (left) and lysotracker (right) at the distal (left) versus the proximal (right) microchannel readout position (Fig. 1). Note how proximal application of PARP1i (orange) on Ctrl MNs (mid gallery) led to virtual distal organelle arrest whereas proximal trafficking remained unaltered (loss of distal straight trajectories compared to Ctrl Mock in top gallery). Conversely, distal application of PARP1i (pink) on Ctrl MNs (mid gallery) had no impact on trafficking on either MFC site, arguing against a local mode of PARPi action. Proximal application of PARGi (green) on FUS MNs (bottom gallery) rescued distal organelle motility (restauration of distal straight trajectories compared to Ctrl Mock in top gallery). Conversely, distal application of PARGi (pale blue) on FUS MNs (bottom gallery) had no beneficial impact on trafficking on either MFC site (unaltered loss of distal straight trajectories compared to FUS Mock in top gallery), arguing against a local mode of PARGi action. Scale bar = 10 μm. **(c)** Multiparametric HC Z-score signatures corresponding to site – specific PARP1i/PARGi treatments in (a) and (b). Site – specific drug application was as indicated in the header. **(d)** Hierarchical cluster dendrogram of entire signatures shown in (c). **(e)** Hierarchical cluster dendrogram of partial signatures comprising either only all distal or proximal parameters (Mitotracker and Lysotracker, respectively) to compare site – specific phenotypes.

Indeed, we document here that (i) chemical disruption of microtubules (nocodazole), ATP production (oligomycin A) or a combination of mitochondria fusion promotor and fission inhibitor (M1 & Mdivi1^14,15^) impacted on the resultant HC signature’s tracking or morphology part (Fig. 2), (ii) mutations in distinct genes (i.e. FUS and TDP43) causing ALS yielded clearly distinct HC signatures (Fig. 3), and finally (iii) that chemical disease modelling using the rescue and mimic compounds recently reported^6^ reverted disease signatures back to a WT-like profile or *vice versa* mimicked the entire FUS profile and not only single parameters (Fig. 4). These findings validate our method as reliable and accurate and open opportunities beyond a purely descriptive characterisation through multiple parameters. Rather, based on the paradigm of modular cell biology^16^, our ultimate goal is to establish a comprehensive data base of HC movie signatures to increase and refine the predictive power of our analytical pipeline. Consistently, our signatures can be assigned to distinct clusters, thereby clearly discriminating between healthy and disease states (Figs 2d, 2e, 3c, 3d, 4d, 4e).

To summarize, because more subtle phenotypes may occur only in minor fractions of the overall organelle population, such accurate and comprehensive multiparametrization requires high throughput organelle tracking in compartmentalized neuronal cultures that enable us to reveal the distal trafficking phenotypes as opposed to the physiological state at proximal sites (Figs 2, 3, 4). For example, even under physiological conditions only 10-20% of mitochondria are processively moving whereas the majority remains stationary^17,18^. This circumstance can easily lead to a bias and erratic results when only a handful of manually selected kymograph profiles are analysed – a still common practice to unravel trafficking patterns in neurites^5,19^.

We provide here the resultant parameter tables of the dynamic organelle tracking (Dynamic Tracking Parameters.xlsx, Data Citation 1) and static morphological analysis (Static Morphology Parameters.xlsx, Data Citation 1) along with the calculated Z-scores (Z-scores.xlsx, Data Citation 1) for the interested reader to test any alternative HC profiling and clustering algorithm. Moreover, we have deposited all movie raw data as 16-bit TIFF image stacks (Movies Data Descriptor – for submission.zip, Data Citation 1) for analysis with any other object recognition and tracking algorithm^20^.

## Methods

General remark: the following sub headers of the methods section mirror the structure of the schematic workflow shown in Fig. 1.

### Choice of cell lines

We choose two different monogenetic ALS causing gene models and respective control to validate the power of our algorithm.

### Generation and differentiation of human iPSC cell lines to MNs in microfluidic chambers (MFCs)

The generation and expansion of iPSC lines from healthy control and familiar ALS patients with defined mutations in the FUS or TDP43 gene was recently described^3,6^. The subsequent differentiation to neuronal progenitor cells (NPC) and further maturation to spinal motor neurons (MN) was performed as described^6,21^. The coating and assembly of MFCs to prepare for the seeding of MNs was performed as described^6^. MNs were eventually seeded for final maturation into one site (Fig. 1, step 4) of MFCs to obtain fully compartmentalized cultures with proximal somas and their dendrites being physically separated from their distal axons as only the latter type of neurite was capable to grow from the proximal seeding site through a microgroove barrier of 900-μm-long microchannels to the distal site. All subsequent imaging in MFCs (Fig. 1, step 5) was performed after 3 weeks of axon growth and MN maturation. Important to note here is, that any compound added to the proximal site for treatment could not diffuse through the microchannels to the distal site due to the higher liquid level there building up a hydrostatic pressure and microflow from distal to proximal site.

### Live imaging of MN in MFCs

To describe ‘gradient’ phenotypes quantitatively for a better understanding of how axonal organelle trafficking increasingly deteriorates with growing distance to their somas, we have established strictly standardized readout windows for live movie acquisition at the distal exit and the proximal entry of the microchannels (Fig. 1, step 5).

To track lysosomes and mitochondria, cells were double-stained with live cell dyes 50 nM Lysotracker Red DND-99 (Molecular Probes Cat. No. L-7528) and 50 nM Mitotracker Deep Red FM (Molecular Probes Cat. No. M22426). Trackers were added directly to culture supernatants and incubated for 1 h at 37 °C. Live imaging was then performed (Fig. 1, step 5) without further washing of cells in the Center for Molecular and Cellular Bioengineering, Technische Universität Dresden (CMCB) light microscopy facility on a Leica HC PL APO 100 × 1.46 oil immersion objective on an inversed fluorescent Leica DMI6000 microscope enclosed in an incubator chamber (37 °C, 5% CO2, humid air) and fitted with a 12-bit Andor iXON 897 EMCCD camera (512 × 512 pixel, 16 μm/pixels on chip, 229.55 nm/pixel at 100x magnification with intermediate 0.7X demagnification in the optical path through the C-mount adapter connecting the camera with the microscope). For more details, refer to https://www.biodip.de/wiki/Bioz06_-_Leica_AFLX6000_TIRF and our recent publication^6^. Fast dual colour movies were recorded at 3.3 frames per second (fps) per channel over 2 min (400 frames in total per channel) with 115 ms exposure time as follows: Lysotracker Red (excitation: 561 nm Laser line, emission filter TRITC 605/65 nm) and Mitotracker Deep Red (excitation: 633 nm Laser line, emission filter Cy5 720/60 nm). Dual channel imaging was achieved sequentially by fast switching between both laser lines and emission filters using a motorized filter wheel to eliminate any crosstalk between both trackers.

Our live set up (at 100x magnification and using the Andor camera as described above) covers in its viewing field at each readout position 2 channels in parallel, each with 117.53 μm of their entire length (900 μm) from either the distal exit or from the proximal entry.

### Site-specific compound treatments of MN in MFCs

In our recent publication^6^, we have observed a remarkable remote mode of action for some compounds which we added exclusively to the proximal soma site. Specifically, when we added the PARG^22,23^ inhibitor Gallotannin (PARGi) exclusively to the proximal site of FUS MNs, all trafficking defects at the distal axon were reverted back to normal Ctrl-like motility. *Vice versa*, PARP1^22,23^ inhibitor ABT-888 (PARP1i) exclusively added to the proximal site of Ctrl MN mimicked all distal trafficking defects of FUS MN axons. Since these alterations at the distal readout occurred in the physical local absence of PARGi or PARP1i, we postulated a long range nucleo^22,23^ - axonal crosstalk. In order to investigate these remote modes of action in more detail and to validate our organelle tracking and morphology analysis (Fig. 1, step 7a, 7b), we included site-specific treatments in our HC profiling as follows:

Validation experiments:

1. **Nocodazole to distal site only in Ctrl MNs**: for local disruption of microtubules and thereby distal organelle motility, control experiment for technical validation of our HC tracking method (Fig. 1, step 7a);
2. **Oligomycin A to distal site only in Ctrl MNs**: for local inhibition of ATP production (through inhibiton of complex V in the respiratory chain of mitochondria) and thereby motor proteins (dyneins and kinesins) for distal organelle motility, control experiment for technical validation of our HC tracking method (Fig. 1, step 7a);
3. **M1 mitochondrial fusion promoter together with Mdivi-1 fission inhibitor to both sites in Ctrl MN**: for augmented fusion of mitochondria to super-elongated organelles^14,15^, control experiment for technical validation of our HC static morphology analysis (Fig. 1, step 7b);

Mimicking and rescue experiments:

1. **PARGi to distal site only in FUS MNs:** no effect^6^;
2. **PARGi to proximal site only in FUS MNs:** rescue of distal organelle trafficking and morphology defects^6^;
3. **PARP1i to distal site only in Ctrl MNs:** no effect^6^;
4. **PARP1i to proximal site only in Ctrl MNs:** mimic of FUS-like distal organelle trafficking and morphology defects^6^;

Gallotannin (Santa Cruz Biotechnology Cat.# sc-202619) was dissolved in water to obtain a 30 mM stock and ABT-888 (Santa Cruz Biotechnology Cat.# sc-202901) was dissolved in DMSO to obtain a 20 mg/ml stock, nocodazole (Sigma-Aldrich Cat.# M1404) and oligomycin A (Sigma-Aldrich Cat.# 75351), M1 fusion promoter (Merk Cat.# 475859) and Mdivi-1 fission inhibitor (Merk Cat.# 4758564) were dissolved in DMSO to obtain a 10 mM stock, respectively. Final concentrations were as follows: 30 μM for PARGi, 2 μg/ml for PARP1i, 10 μM for nocodazole, oligomycin A, M1 and Mdivi-1, respectively. DMSO was used for Mock controls (0.1%). PARGi and PARP1i were added to distal or proximal site in MFCs 72 h prior to imaging (Fig. 1, step 5) due to the slower response via the remote nucleo-axonal crosstalk, M1 and Mdivi-1 were added together to both sites 24 h prior to imaging (Fig. 1, step 5), nocodazole and oligomycin A were added to the distal site 4 h prior to imaging (Fig. 1, step 5) owing to their rapid local action and to avoid prolonged toxicity as well as their leaking to the proximal site due to the microflow from distal to proximal.

To summarize, by comparing the distal *versus* the proximal HC response upon site-specific compound addition, we aimed to clearly distinguish between local and remote action as well as carefully assessing the leakiness of the MFC’s microgroove barrier.

### Handling of meta data throughout the work flow

Original file containers generated by the microscope software were opened in FIJI and channels for Mito- and Lysotracker separated. Individual movies were saved as 16 bit TIFF image stack files and subsequently annotated with information regarding the experimental conditions (Fig. 1, step 6), e.g. ‘Mock-treated Ctrl cells imaged with Lysotracker at distal readout, Movie #1’. To this end, we sorted each movie into a prepared hierarchical folder tree (Fig. 1, step 6, Movies Data Descriptor – for submission.zip, Data Citation 1) in which the file path encoded its specific experimental condition, thereby straightforwardly realizing its annotation in a multi user environment and enabling the subsequent data mining. For this report we provide 5 representative movies for each condition and cell line (Movies Data Descriptor – for submission.zip, Data Citation 1).

### Automated object recognition and dynamic tracking with the FIJI Track Mate plugin

Movies sorted into the hierarchical folder tree (Movies Data Descriptor – for submission.zip, Data Citation 1) were opened individually and movement of organelles was analysed (Fig. 1, step 7a) by initial object recognition and subsequent linking of these objects between different time points (i.e. image frames). The Track Mate plugin v2.7.4 provided by FIJI was utilized to calculate dynamic tracking parameters (e.g. organelle mean speed and track displacement). Pixel calibration of parameters was used as provided by the microscope to convert pixel into μm dimensions and frame # into seconds. The object recognition tool used a detector based on the difference of Gaussians with an estimated object diameter of 1.6 μm and enabled sub pixel localization. The quality threshold was adjusted to 45 for each video. Linking was achieved with the Linear motion LAP tracker (Kalman tracker) and initial search distance of 2 μm and gap distance of 2 frames. Finally, a duration filter of 3 s was added to only incorporate tracks that were at least present for 10 consecutive images. For a full list of all 12 obtained parameters refer to Dynamic Tracking Parameters.xlsx, Data Citation 1. Please note that we used only 9 of these in the final Z-score signatures (Fig. 1, step 10), because all other parameter were of no biological relevance. Thus, in Dynamic Tracking Parameters.xlsx, Data Citation 1, these 9 selected parameters were highlighted in brown, whereas parameters not considered in this report were greyed out on the right. The results of the Track Mate analysis were saved as one CSV - file per movie in the folder of the original movie (Fig. 1, step 8).

### Automated object recognition and static morphology analysis with the FIJI Morphology macro

The movies were automatically loaded consecutively from the parental folder (Movies Data Descriptor – for submission.zip, Data Citation 1) into FIJI with our custom-tailored morphology macro (Fig. 1, step 7b) (Morphology-macro.ijm.txt, Data Citation 1). Only the first movie frame was extracted for object recognition (either mitochondria or lysosomes) using the auto threshold and analyze particle function. Measurement of each detected object returned a whole set of morphological organelle shape descriptors such as object area, diameters of outer and inner circumference (Feret diameters), integral fluorescence intensity, circularity and the ratio of the major/minor radius of eclipses fitted to objects (i.e. the aspect ratio). For a full list of all obtained parameters refer to Static Morphology Parameters.xlsx, Data Citation 1. Please note that we used only the lysosmal outer Feret diameter and the mitochondrial aspect ratio in the final Z-score signatures (Fig. 1, step 10), because all other readouts were not reflecting relevant biological parameters or simply linearly depending on the selected ones. Thus, in Static Morphology Parameters.xlsx, Data Citation 1, the Feret diameters and aspect ratios were highlighted in brown whereas parameters not considered in this report were greyed out on the right.

The results were saved as one CSV – file per movie in the folder where the movie was stored (Fig. 1, step 8). Therefore, each movie in the hierarchical folder tree obtained 2 CSV files - one from the Track Mate and another from the morphology macro.

### Data mining in CSV result files and assembly of final EXCEL result tables with KNIME

The ‘Konstanz Information Miner’ (KNIME) was used to process, filter and group the data (Fig. 1, step 9) obtained from both movie analyses conducted in FIJI (dynamic tracking and static morphology). The established workflow is available online (see section ‘Code availability’) and can be adjusted according to the user’s preference. In short, the core workflow contains one segment for data loading, one for filters and calculations and lastly the export of agglomerated data. The meta data (i.e. the annotated information about the experimental condition) that will be assigned to each CSV - file is extracted from the folder tree, where the file is stored. The default table header layout is as follows: “X:\Project\Experiment\Marker\Readout position\Treatment\Disease\Line\”. Each row in the final result tables (Dynamic Tracking Parameters.xlsx, Static Morphology Parameters.xlsx, Data Citation 1) refers to a single track or organelle from the movie specified under ‘Filename’ with all its elucidated parameters in the right table part. Exact definition of each experimental folder layer enables the user to pool the data later on different desired levels, depending on the investigated context. Nodes that have to be modified for each individual user are marked by a yellow rectangle. We have implemented a row filter node by the end of the workflow to exclude all tracks with a displacement below 1.2 μm to remove non-processive jitter. The final XLS Writer node is exporting the filtered result table as XLSX table (Dynamic Tracking Parameters.xlsx, Static Morphology Parameters.xlsx, Data Citation 1).

### Assembly of phenotypic HC signatures

From both final parameter result tables (Fig. 1, step 9) we calculated the mean and standard deviation (SD) for each parameter (nine dynamic tracking parameter and one static morphology parameter) over the entire batched organelle population of each condition (i.e. movie pool of each condition, e.g. Lysotracker\Distal\Mock\Ctrl\all movies) and expressed its normalized deviation (Z-score) from Ctrl Mock at the proximal readout (i.e. the baseline) using the equation

Z(PxC)=PxC¯−PxCtrl¯SDxCNxC+SDxCtrlNxCtrl

with

$Z(P_{x}^{C})$: Z-score of parameter x (e.g. mean organelle speed) at condition C (e.g. FUS Mock Distal Lysotracker);

$\overset{¯}{P_{x}^{C}}$: mean of parameter x at condition C;

$\overset{¯}{P_{x}^{Ctrl}}$: mean of parameter x at Ctrl Mock proximal;

$SD_{x}^{C}$: standard deviation of parameter x at condition C;

$SD_{x}^{Ctrl}$: standard deviation of parameter x at Ctrl Mock proximal;

$N_{x}^{C}$: sample size of parameter x at condition C;

$N_{x}^{Ctrl}$: sample size of parameter x at Ctrl Mock proximal;

To obtain the final phenotypic HC signatures, Z-scores were plotted for each condition (e.g. FUS Mock) over all parameters in the following order:

For Mitotracker at the distal readout, parameter # 1-10:

1. Track Duration
2. Track Displacement
3. Track Mean Speed
4. Track Max Speed
5. Track Min Speed
6. Track Median Speed
7. Track SD Speed
8. Track Length
9. Ratio Antero-/Retrograde Tracks
10. Mitochondrial Aspect Ratio (ratio of major/minor radius of fitted ellipse)

The same parameter set applied for Mitotracker at the proximal readout, resulting in parameter # 11-20.

The same parameter set applied for Lysotracker at the distal readout, resulting in parameter # 21-30, except that the aspect ratio was replaced by the Feret diameter as size measure (parameter # 30, diameter of outer circumference fitted to object) as lysosomes always appeared of globular morphology.

The same parameter set applied for Lysotracker at the proximal readout, resulting in parameter # 31-40.

Z-scores.xlsx, Data Citation 1, provides all Z-scores used to plot the phenotypic HC signatures shown in Fig. 1 (step 10), Fig. 2c, 3b, and 4c. Parameter # 9 (ratio antero-/retrograde tracks) was not returned directly by the FIJI Track Mate plugin (Fig. 1, step 7a) but calculated afterwards as per movie parameter as follows: the last tracking parameter, i.e. track angle (expressed as radian measure) was used for a range check to classify each track as either anterograde or retrograde. Since a track angle of zero equates to a horizontal vector pointing retrogradly to the proximal right site and π to the distal left site, a track angle between - π/2 and π/2 was classified as retrograde track. Conversely, a track angle below - π/2 or above π/2 was classified as anterograde track (Dynamic Tracking Parameters.xlsx, Data Citation 1, far right columns). For each movie, the ratio antero-/retrograde tracks was then simply calculated by dividing the anterograde by the retrograde track count. The Z-score of this parameter was calculated as for all other parameters but on a per movie instead on a per track or per organelle base.

Finally, to validate our batched movie analysis, we sought to obtain an estimate of the intra- vs inter-experimental variability. To this end, we compared the variabilities of (i) the organelle population of one channel vs the other within a given movie, (ii) one movie vs another within a given experiment, and (iii) one movie of experiment 1 vs another of experiment 2. The resultant pairwise Z-scores were marginal (mostly less than 10%) as compared to our stringent significance threshold (Z-score ≥ 5 or ≤ −5, highlighted by grey horizontal lines e.g. in Fig. 2c, see also Technical Validation, section ‘Validation through drug manipulations’). Therefore, the signatures presented in our manuscript are robust common denominators deduced from pooled data, thereby enabling a global HC phenotypic assessment of different lines and treatments.

### Clustering of phenotypic signatures

While Z-scores served as measure for the degree and significance of deviation from ctrl proximal conditions for individual parameters, we wished to assess our multiparametric signatures as a whole with respect to similarities and deviations. This has the advantage of discriminating between disease and physiologic states more objectively, as comprehensive consideration of multiple parameters is less prone to possible noise and bias of individual parameters. To this end, we performed unsupervised clustering of the multiparametric data sets in KNIME. To avoid high dependencies on single parameters, normalization of data is necessary prior to clustering, a requirement already fulfilled with the parameter Z-score calculation above. Thus, we used the entire Z-score signature of all parameters above for clustering in KNIME with the “Hierarchical clustering” node, which uses a bottom-up strategy. We used the Euclidean average distance to agglomerate data and calculate the dendrograms (Figs 2d, 3c, 4d).

### Code availability

The code of our customized FIJI Morphology macro was deposited in figshare as Morphology-macro.ijm.txt, Data Citation 1.

The code of our default KNIME workflow was deposited in figshare as KNIME workflow.zip, Data Citation 1.

The Track Mate plugin is preinstalled in newer versions of FIJI (64 bit) and can be obtained from https://imagej.net/Category:Plugins

## Data Records

1. All movie raw data (16-bit TIFF image stacks) were sorted into the hierarchical folder tree and are deposited in figshare (Movies Data Descriptor – for submission.zip, Data Citation 1).
2. The XLSX result table of dynamic tracking parameters is available in figshare as Dynamic Tracking Parameters.xlsx, Data Citation 1. For details on how it was generated, refer to Methods, subsections ‘Handling of meta data throughout the work flow’, ‘Automated object recognition and dynamic tracking with the FIJI Track Mate plugin’, ‘Data mining in CSV result files and assembly of final XLSX result tables with KNIME’. On the left, highlighted in blue, Dynamic Tracking Parameters.xlsx, Data Citation 1, shows selected meta data information for each individual track in 4 columns as specified by the file path. Further layers of information could easily be added by further folder levels and configuring the respective nodes of the KNIME workflow. The blue part defines the experimental condition of each track and provides full transparency from the original movie file via the individual track (mid part in black showing filename and Track ID) to the final outcome of the tracking analysis in FIJI Track Mate, i.e. the parameters on the right highlighted in brown. Parameters returned by Track Mate but not further considered are greyed out on the far right.
3. The XLSX result table of static morphological parameters is available in figshare as Static Morphology Parameters.xlsx, Data Citation 1. Same generation and layout as Dynamic Tracking Parameters.xlsx, Data Citation 1, except that the FIJI Morphology macro was used, for details refer to Methods, subsection ‘Automated object recognition and static morphology analysis with the FIJI Morphology macro’.
4. The XLSX Z-score table used to plot all Z-score signatures as shown in Fig. 2c,3b and 4c is available in figshare as Z-scores.xlsx, Data Citation 1.
5. The illustration of the axon–specificity in our readout windows is available in figshare as Axon Specificity.jpg, Data Citation 1.
6. The illustration of the object recognition and tracking analysis is available in figshare as Tracking Illustration.jpg, Data Citation 1.

## Technical Validation

### Validation through drug manipulations

In order to test the sensitivity of our multiparametric readout with respect to local, site-specific (i.e. distal *versus* proximal) changes in organelle motility and shape, we tested the Z-score HC signature response in Ctrl MNs upon addition of compounds knowingly impacting on trafficking and mitochondria morphology (Fig. 2). Specifically, we used (i) nocodazole to depolymerize and disrupt microtubule and therefore transport^24^, (ii) oligomycin A to block mitochondrial ATP production through complex V ATP synthase inhibition^25^, which leads to slowing down of dyneins and kinesins and (iii) a mixture of M1 fusion promoter^15^ and Mdivi-1 fission inhibitor^14^ to cause an extended elongation of mitochondria. Following common statistical standards, we regarded Z-score values of 1 only as theoretical border line significance and considered a more stringent minimum of 5 as clearly significant deviation from Ctrl conditions (Fig. 2c, marked Z-score range from -5 to 5). As expected, Ctrl Mock (blue signature, Fig. 2c) exhibited only marginal deviations at the distal site as compared to the proximal baseline (Z = 0) for either type of organelle (left: mitochondria, right: lysosomes, Fig. 2c), consistent with unaltered, physiological trafficking in distal axons. By contrast, addition of either nocodazole or oligomycin A excusively to the distal site (Fig. 2a) caused a reduction in organelle motility already visible in the maximum intensity projections of the movie stacks (Fig. 2b) and, consistently, a highly significant negative deviation of most parameters at the distal site, (particularly mean, max and median speed) whereas the proximal signature parts remained mostly unaltered (Fig. 2c, pink and black signatures), consistent with local disruption of organelle motility and tight adherence of the MFC (Fig. 2a). However, lysosomes exhibited proximal parameter deviations weakly reminiscent (Z ~ 5) of the distal signature part for oligomycin A (Fig. 2c, pink signature) suggesting some little leakiness due to the micro flow from distal to proximal within the incubation time of 4 h. Alternatively, oligomycin A could have been actively transported intracellularly from distal to proximal by directed axoplasmic flow.

As for organelle - specific features in the mitochondrial (left part) *versus* the lysosomal (right part) of the signature, both types of organelle responded mostly in a similar fashion to nocodazole and oligomycin A treatment (Fig. 2c, pink and black signatures, distal parts) while showing some subtle differences. For example, distal lysosomal track displacement was significantly reduced (Fig. 2c. parameter # 22, Z ≤ -5) whereas distal mitochondrial track displacement was hardly altered or even increased (Fig. 2c, parameter # 2, Z ≤ 5). This difference presumably illuminates on the distinct nature of either type of organelle. Feasibly, as lysosomes move much faster in a more processive fashion^6,26–28^ (Dynamic Tracking Parameters.xlsx, Data Citation 1), disruption of their motility through nocodazole or oligomycin A impacts relatively more on their track displacement (measure for processivity, i.e. straightness of movement) whereas physiological mitochondria exhibit a major stationary fraction anyway^17,18^.

As for drug – specific features, the response to either type of treatment was, again, largely the same but with some subtle differences, thereby reflecting on the distinct modes of action of nocodazole (direct disruption of the underlying microtubule tracks) *versus* oligomycin A (indirect energy deprivation of motor proteins). For example, nocodazole had no impact on lysosomal mean speed, led to increased min speed and decreased track length (Fig. 2c, parameters # 23, 25, 28, black signature). Conversely for oligomycin A, distal lysosomal mean and min speed were both significantly reduced (Fig. 2c, parameters # 23, 25, pink signature) whereas their track length was hardly decreased, suggesting that motor activity was slowed down due to the ATP deprivation but straight, processive movement still occurred at lower speed as microtubules remained intact. This differential drug response of lysosomes was not overt in the mitochondrial counterpart of the signature, again illuminating on the different organelle identity and their trafficking patterns, feasibly due to different underlying motors and their regulation^29,30^. However, few parameters evaded a plausible biological interpretation as their peculiar deviation seemed to contradict the overall gross effect on microtubule-dependent organelle motility expected upon treatment with nocodazole. Specifically, the min speed upon nocodazole treatment was increased in Ctrl cells for distal lysosomes (Fig. 2c, parameter # 25, black signature) and even more for distal mitochondria (Fig. 2c, parameter # 5, black signature). A similar observation was made for distal mitochondria in mutant FUS Mock (Fig. 3b, parameter # 5, red signature). Two possible explanations exist: first, and most likely, the majority of the organelle population was more stationary and removed through the post filtering thresholds (track duration ≥ 3 s, track displacement ≥ 1.2 μm, see Methods), thereby weighting the mean values for min speed of the remaining escapers more profoundly. Second, less probable, the particular acute treatment conditions for nocodazole and oligomycin A could have caused some additional parameter variability: due to the known toxicity of these agents, the image acquisition had to be started after a brief incubation time to obtain results before they became too toxic. Therefore, a concentration boundary was probably migrating from distal through the readout window during the image session, thereby determining that the effective concentration and cellular response followed an unknown underlying spatial-temporal-dependent pattern. These complex kinetics were likely to be different in each microchannel, depending on the steric thickness of each axon bundle, etc. However, the overall signature responses also in fast acting compounds (such as nocodazole or oligomycin A in our case) were still very robust and significant for the majority of all parameters, thereby arguing in favor of these validators as the overt differential drug response demonstrated the high analytical resolution of our HC profiling. Thus, the noted variability with stronger impact on the average min speed compared to others made this parameter less reliable. However, this also clearly underpins the robustness of our approach of focusing on entire multiparametric HC signatures rather than the biological interpretation of single parameters, which might be impaired under certain conditions described above. To summarize the dynamic trafficking response, our multiparametric readout detected the distinct, local drug actions as well as the organelle - specific trafficking parameters. We were able to identify subtle differences of the resultant Z-score signatures while at the same time highlighting overt phenotypes induced by either the disruption of microtubules or energy deprivation of all motor proteins.

Furthermore, we included static morphology parameters in our signature to assess organelle shape (Fig. 2c, parameters # 10, 20, 30, 40). These parameters were assessed only in the first image of the stack with our FIJI Morphology macro. Validation was achieved by treating Ctrl MNs on both MFC sites with a mixture of M1 fusion promoter^15^ and Mdivi-1 fission inhibitor^14^ (Fig. 2a) to promote elongation of mitochondria. Already in the raw images, an extended elongation of mitochondria at both the distal and proximal site became indeed clearly overt (Fig. 2b). Consistently, parameters # 10 and 20 in the corresponding signature increased significantly (Fig. 2c, green signature, Z ~ 10 for the distal and proximal aspect ratio). In addition, some side effects on proximal mitochondrial speed parameters occurred (Fig. 2c, green signature, parameters # 13 - 17) which were, however, of no relevance for the technical validation here. In conclusion, our multiparametric HC readout was sensitive in detecting reliably alterations in dynamic trafficking patterns as well as in static organelle shape.

While the Z-score indicates to what extend a single parameter deviates from control conditions, we strived to have an objective measure to compare entire mutliparametric signatures and group them based on similarities. We generated a hierarchical cluster dendrogram with KNIME (Fig. 2d). Nocodazole and oligomycin A were assigned to a cluster distinct to control Mock and M1 & Mdivi-1, confirming that both treatments led to a similar strong response over control Mock with a group difference Z-score > 60 compared to control and M1&Mdivi (this hierarchical clustering Z-score is not to be mistaken with the individual parameter Z-score, see Methods, subsection ‘Clustering’). Because M1 & Mdivi had only an impact on the static parameters, they were grouped with Ctrl Mock at a smaller Z score difference. Thus, the unsupervised clustering detected the different modi of action and higher impact strenghtes of nocodazole/oligomycin A against M1 & Mdivi-1 over Mock treatment, thereby confirming the apparent differences between the signatures (Fig. 2c) and enabling clear grouping.

Finally, we further refined the cluster analysis with partial signatures comprising either only all distal or proximal parameters (Mitotracker and Lysotracker, respectively) to compare site – specific phenotypes (Fig. 2e). Oligomycin A (pink) and nocodazole (black) at the distal readout agglomerated in a distinct cluster on the right due to their strong local action (Fig. 2e) whereas their nearly unaltered proximal counterparts clustered more closely to the physiological Ctrl Mock (blue, distal and proximal readout). The proximal part of M1 & Mdivi-1 treatment (green) was slightly more deviant to the Ctrl Mock parts (blue) as compared to its distal counterpart (green) due to the visible side effects in its corresponding proximal signature part in (c) (Fig. 2e).

### Validation by distinguishing different disease phenotypes

Having validated our multiparametric readout through drug manipulations in healthy control MNs, we next tested our analytical pipeline (Fig. 1) on disease models with confirmed defects in axonal trafficking. We and others recently reported distal organelle arrest in spinal MN of FUS^5,6^ and TDP43 mutants^3^ characterized by a reduction in track mean speed and displacement. These trafficking perturbations became already apparent in the maximum intensity projections of the movie raw data, particularly for FUS at the distal site (Fig. 3a). The corresponding FUS signature exhibited strong negative deviations of most speed parameters for either organelle types along with shortening of mitochondria (parameter # 10) at the distal site. The proximal site remained mostly physiological (Fig. 3b, red signature). TDP43 and FUS displayed both only marginal alterations at their respective proximal sites but were clearly distinct in their distal signature parts (Fig. 3b, red and green signature). For example, the reduction in max speed was less pronounced in TDP43 (parameters # 4, 24) along with an almost unaltered speed variation in the tracks (SD speed, parameters # 7, 27). The observed lower speed and track displacement suggest residual organelle motility in TDP43 (parameters # 2, 12) while FUS exhibited a pronounced organelle arrest. (Fig. 3b, red and green signature). The distinct and more severe deviation of the FUS mutant to Ctrl MNs was further confirmed by hierarchical clustering (Fig. 3c): with a group difference of Z > 28 TDP43 clustered closer with Ctrl than with FUS. Further refined clustering with partial signatures (Fig. 3d) (only distal and proximal subparts) revealed that both physiological Ctrl Mock parts (blue, distal and proximal) clustered closely with the proximal FUS Mock part (red) on the right due to the close physiological trafficking state whereas the drastic organelle arrest in the distal FUS Mock part on the far left (red) was highly distinct to the physiological parts at the proximal site (Fig. 3d). TDP43 showed some moderate deviation in its proximal part (green) from the physiological clusters (Ctrl Mock, blue, and FUS Mock proximal, red) and a clear deviation in its distal part (green), albeit less drastic than FUS Mock (red). Recently, we documented a global trafficking defect in mutant TDP43^3^ based on the analysis of only two parameters (i.e. mean speed and track displacement). However, in this study we took the whole multiparametric TDP43 signature into account and obtained a more accurate refinement of our original finding by uncovering a global phenotype with a profound distal emphasis (Fig. 3d).

### HC imaging as powerful tool for pathway or drug validation

Our system can be used to cross validate intervention strategies and elucidate involved pathways. Application of a PARP1 inhibitor to the proximal soma site of MFCs (Fig. 4a) led to a mimic of FUS-like trafficking defects at the distal axonal site, whereas trafficking at the proximal site remained unaltered^6^ (Fig. 4b, c, orange label/signature). Conversely, site-specific distal application of PARP1i (Fig. 4a) did not perturb trafficking at all^6^ (Fig. 4b, c, pink label/signature), showing it’s mediation through nuclear FUS localization^22,23^.

Site-specific inhibition of the PARP1 antagonist PARG^23^ (Fig. 4a) prevents the cleavage of the polyadenylribosyl moiety from FUS, thereby prolonging the retention time of FUS at nuclear DNA damage sites^23^. Treatment with PARG inhibitor rescued FUS recruitment in nuclear DNA Laser ablation experiments^6^ and was likewise able to rescue the distal axonal trafficking phenotype when applied to proximal sites of MFCs^6^ (Fig. 4a-c, green label/signature). Conversely, PARGi incubation at distal MFC sites did not alter the disease status (Fig. 4a-c, pale blue label/signature). These results further evidenced the tight adherence and strengthen the view of a remote nucleo – axonal crosstalk as they argue against a local mode of action on trafficking.

The efficacy of the proximal drug application against the failure of distal application in mimicking (PARP1i) or rescuing (PARGi) the distal FUS phenotype was further confirmed by hierarchical clustering (Fig. 4d). Two main Clusters were observed that indicated either a healthy status containing Ctrl Mock, FUS PARGi to proximal and Ctrl PARP1i to distal, or a diseased status containing FUS Mock, FUS PARGi to distal and Ctrl PARP1i to proximal. Within the ‘diseased cluster, proximal PARP1i application to Ctrl formed a subcluster with FUS Mock against the slightly more distant distal PARGi application to FUS (Fig. 4d, right main cluster). This was possibly due to minor local side effects of the latter on trafficking (Fig. 4c, compare pale blue against dark blue signature in the lysosomal part).

Further refined clustering with partial signatures (distal and proximal parts only) (Fig. 4e) yielded a main ‘pathological’ cluster on the right that comprised the natural distal FUS Mock defects (red) along with its inefficient distal treatment with PARGi (pale blue) and its efficient mimic due to proximal treatment with PARP1i on Ctrl MNs (orange). Conversely, all other conditions agglomerated more closely in the ‘physiological’ main cluster on the left (blue, Ctrl Mock distal and proximal) (Fig. 4e). As for the proximal readouts, this was because trafficking remained always physiological in FUS or Ctrl regardless of Mock or treatment. As for the distal readouts, the efficient rescue in FUS through proximal PARGi addition (green) or the inefficient treatment in Ctrl MNs with distal PARP1i addition (pink) rendered these conditions physiological (Fig. 4e).

To summarize, our multiparametric HC profiling was very sensitive in detecting remote drug actions through axons across the MFC microgroove barrier against local modi of actions. Therefore, our method provides a powerful analytical tool to (i) detect the effects of compounds/genetic interrogation on the full content signature thus allowing better predictability of partial vs. complete effects and (ii) further interrogate the intriguing nucleo – axonal crosstalk with more compounds and RNAi reagents for mechanistic dissection.

## Additional information

**How to cite this article**: Pal, A. *et al.* High content organelle trafficking enables disease state profiling as powerful tool for disease modelling. *Sci. Data*. 5:180241 doi: 10.1038/sdata.2018.241 (2018).

**Publisher’s note**: Springer Nature remains neutral with regard to jurisdictional claims in published maps and institutional affiliations.

## Supplementary Material
